# Lung volume dependence of respiratory function in rodent models of diabetes mellitus

**DOI:** 10.1186/s12931-020-01334-y

**Published:** 2020-04-09

**Authors:** Roberta Südy, Álmos Schranc, Gergely H. Fodor, József Tolnai, Barna Babik, Ferenc Peták

**Affiliations:** 1 Department of Medical Physics and Informatics, University of Szeged, 9 Koranyi fasor, Szeged, H-6720 Hungary; 2 Department of Anaesthesiology and Intensive Therapy, University of Szeged, 6 Semmelweis Street, Szeged, H 6725 Hungary

**Keywords:** Forced oscillations, Hyperglycemia, Respiratory mechanics, Airway responsiveness, Tissue viscoelasticity

## Abstract

**Background:**

Diabetes mellitus causes the deterioration of smooth muscle cells and interstitial matrix proteins, including collagen. Collagen and smooth muscle cells are abundant in the lungs, but the effect of diabetes on airway function and viscoelastic respiratory tissue mechanics has not been characterized. This study investigated the impact of diabetes on respiratory function, bronchial responsiveness, and gas exchange parameters.

**Methods:**

Rats were allocated randomly to three groups: a model of type 1 diabetes that received a high dose of streptozotocin (DM1, *n* = 13); a model of type 2 diabetes that received a low dose of streptozotocin with a high-fat diet (DM2, *n* = 14); and a control group with no treatment (C, *n* = 14). Forced oscillations were applied to assess airway resistance (Raw), respiratory tissue damping (G), and elastance (H). The arterial partial pressure of oxygen to the inspired oxygen fraction (PaO_2_/FiO_2_) and intrapulmonary shunt fraction (Qs/Qt) were determined from blood gas samples at positive end-expiratory pressures (PEEPs) of 0, 3, and 6 cmH_2_O. Lung responsiveness to methacholine was also assessed. Collagen fibers in lung tissue were quantified by histology.

**Results:**

The rats in groups DM1 and DM2 exhibited elevated Raw, G, H, and Qs/Qt, compromised PaO_2_/FiO_2_, and diminished airway responsiveness. The severity of adverse tissue mechanical change correlated with excessive lung collagen expression. Increased PEEP normalized the respiratory mechanics, but the gas exchange abnormalities remained.

**Conclusions:**

These findings indicate that diabetes reduces airway and lung tissue viscoelasticity, resulting in alveolar collapsibility that can be compensated by increasing PEEP. Diabetes also induces persistent alveolo-capillary dysfunction and abnormal adaptation ability of the airways to exogenous constrictor stimuli.

## Introduction

Uncontrolled diabetes mellitus (DM) can result in long-term hyperglycemia that activates various molecular pathways [1], leading to inflammation [2], endothelial [3, 4], smooth muscle cell dysfunction [5], and protein destruction [6]. These mechanisms involve the formation of advanced glycation end products (AGEs) [7], which cause deterioration to the structure and function of matrix proteins and affect matrix–matrix and matrix–cell interactions [8].

The pathophysiological consequences of DM may also damage the pulmonary endothelium and bronchial smooth muscle cells, so the lungs can be among the organs most affected by DM [9–12]. Endothelial dysfunction contributes to the remodeling of the airways and lung parenchyma related to oxidative stress and the overproduction of reactive oxygen species [13–15]. These processes manifest as the sustained contraction of bronchial smooth muscle cells [13, 14, 16, 17], thickening of the alveolar walls [10, 18], and changes to the elastin–collagen matrix in the lung parenchyma [10, 19, 20]. Hyperglycemia also affects type II pneumocyte cells, resulting in decreased surfactant biosynthesis and secretion [21, 22].

Conflicting results have been reported for the effects of DM on lung function. Some studies have demonstrated declines in spirometry outcomes [23–26], whereas others found no detectable change [27–30]. Possible explanations for this discrepancy include the dependence of spirometry results on the effort and cooperation of patients [31, 32], and substantial differences between study populations, such as the type and severity of DM, age, and comorbidities. Furthermore, forced expiratory parameters have limited sensitivity to detecting detrimental changes in lung tissue and peripheral airway mechanics [9, 27, 30], the aspects of the lung affected primarily by DM [30, 33]. Thus, it remains unclear how DM affects airway function and the viscoelastic mechanics of respiratory tissue, including its dissipative and elastic properties. In particular, it is unknown whether DM influences the changes in mechanical parameters that occur with changes in lung volume.

This study aimed to characterize changes in the airway and respiratory tissue viscoelastic parameters to clarify the pulmonary consequences of hyperglycemia. Therefore, we applied a measurement technique that allowed the exact characterization of these changes in well-established animal models of type 1 and type 2 DM. We hypothesized that DM would result in the development of compromised airway function and changes in airway responsiveness resulting from the sustained contractile response of the bronchial smooth muscle, as well as deterioration to both the dissipative and elastic components of respiratory tissue viscoelasticity. We also assessed the contribution to the DM-induced changes in lung function of the loss of lung volume, gas exchange abnormalities, proliferation, and fiber–fiber interaction by evaluating changes in the functional residual capacity (FRC), the arterial partial pressure of oxygen to the inspired oxygen fraction (PaO_2_/FiO_2_), intrapulmonary shunt (Qs/Qt), and lung histology.

## Materials and methods

### Ethical considerations

This study was approved by the National Food Chain Safety and Animal Health Directorate of Csongrád County, Hungary (no. XXXII./2098/2018), on September 24, 2018. The procedures were implemented in compliance with the guidelines of the Scientific Committee of Animal Experimentation of the Hungarian Academy of Sciences (updated Law and Regulations on Animal Protection: 40/2013. [II. 14.], the Government of Hungary), and European Union Directive 2010/63/EU on the protection of animals used for scientific purposes. The results were reported in line with the ARRIVE guidelines.

### Inducing diabetes

**Fig. 1:**
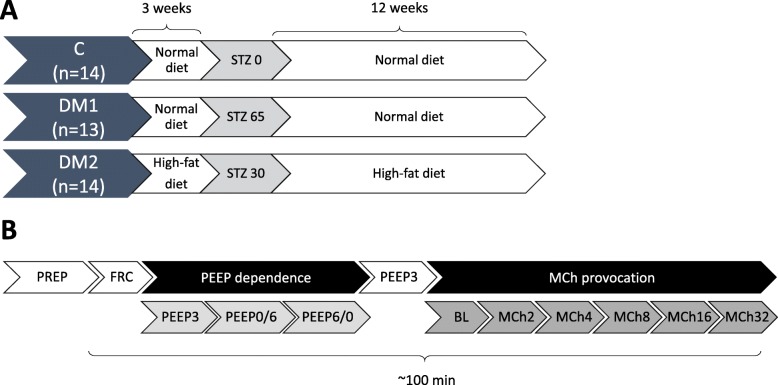
Schemes summarizing the pretreatment strategies (**a**) and the experimental protocol (**b**). Forced oscillation measurements and blood gas analyzes were performed at three different positive end-expiratory pressures (0, 3, and 6 cmH_2_O: PEEP0, PEEP3, and PEEP6, respectively). Forced oscillation respiratory mechanics were assessed at a PEEP of 3 cmH_2_O during provocation tests with increasing doses (2–32 μg/kg/min) of methacholine (MCh) following the baseline measurement (BL). Abbreviations: DM1, model of type 1 diabetes; DM2, model of type 2 diabetes; C, control group; STZ, streptozotocin in mg/kg; FRC, measurement of functional residual capacity; PREP, animal preparations

The study used 5-week-old male Wistar rats (mean weight ± 95% confidence interval, 187.3 ± 3.7 g, *n* = 42). During the initial phase of the study protocol, the animals were housed for 15 weeks under close daily observation in accordance with the animal welfare assessment and 3R guidelines. Well-validated models were adapted to induce models of type 1 and type 2 DM, with proven hyperglycemia, insulin resistance, and the diffuse degeneration of pancreatic cells [34–39]. The 5-week-old rats were assigned randomly to three protocol groups: model of type 1 DM (DM1 group, *n* = 14), model of type 2 DM (DM2 group, n = 14), and control group (n = 14). They were fed in accordance with their group allocations (Fig. 1a): rats in the DM1 and control groups received a normal diet (fat and protein contents of 3.9 and 20.1%, respectively), whereas those allocated to the DM2 group were fed a high-fat diet (Altromin C1080, 47% fat, 18% protein, 35% carbohydrate; Altromin Spezialfutter GmbH & Co. KG, Lage, Germany). After a 3-week period (at the age of 8 weeks), the DM1 group rats were treated with a single high dose of streptozotocin (STZ, 65 mg/kg) [34, 38], the DM2 group rats were treated with a low dose (30 mg/kg) of STZ [35–37, 39], and the controls received the vehicle of the STZ (citrate buffer, pH 4.4). Before the injection of STZ or vehicle and 1 week after the treatment (age of 9 weeks), fasting glucose levels were measured from the tail vein using an Accu-Chek Active blood glucose meter (Roche, Basel, Switzerland). Four rats in the DM2 group were administered a second injection of 30 mg/kg STZ treatment because their fasting glucose level was < 7.8 mmol/l [36, 39]. One animal in the DM1 group was sacrificed after 7 weeks due to isolation and its unsatisfactory health condition.

### Animal preparation

The experiments were performed on week 16 (age of 20 weeks; Fig. 1b). Anesthesia was induced by an intraperitoneal injection of sodium pentobarbital (45 mg/kg; Sigma-Aldrich, Budapest, Hungary). After administering local anesthesia (subcutaneous lidocaine, 2–4 mg/kg), a polyethylene cannula with an inner diameter of 18G was inserted into the trachea through a tracheostomy. The tracheal cannula was connected to a small animal ventilator (Model 683; Harvard Apparatus, South Natick, MA, USA), and the animal was mechanically ventilated (55–60 breaths/min, tidal volume 7 ml/kg, with a fraction of inspired oxygen [FiO_2_] of 21%). The femoral artery was catheterized for blood pressure measurements and the collection of blood samples. The femoral vein was cannulated for drug administration and the jugular vein was secured for drug administration and the collection of venous blood samples. Anesthesia was maintained with pentobarbital (5 mg/kg, intravenous (iv.), every 30 min). The rat was placed on a heating pad (model 507223F; Harvard Apparatus, Holliston, MA, USA) and its body temperature was maintained at 37 ± 0.5 °C. The forced oscillation measurements to evaluate the impedance of the respiratory system were made under neuromuscular blockade, achieved by the repeated iv. administration of pipecuronium (0.1 mg/kg every 30 min; Arduan, Richter-Gedeon, Budapest, Hungary).

### Functional residual capacity

The FRC was measured as described previously [40]. Briefly, the rat was tracheostomized and placed in a whole-body plethysmography box. The trachea and box were closed at end-expiration and the measurements were made while the animal generated breathing efforts against the closed trachea. The FRC was calculated from the simultaneously measured tracheal and box pressure signals by applying the Boyle–Mariotte law [40].

### Airway and respiratory tissue mechanics

The input impedance of the respiratory system (Zrs) was measured by the forced oscillation technique, as described previously [41]. Briefly, the tracheal cannula was connected to a loudspeaker-in-box system, while ventilation was suspended at end-expiration. A small-amplitude pseudorandom signal (< 1.5 cmH_2_O, with 23 non-integer multiple-frequency components between 0.5 and 20.75 Hz) was applied to the tracheal cannula through a wave tube (polyethylene; length 100 cm, internal diameter 2 mm). To maintain uniform transpulmonary pressure during the measurements, the pressure in the loudspeaker box was set to be equivalent to that of the positive end-expiratory pressure (PEEP) used for that experiment (0, 3, or 6 cmH_2_O). During 8-s measurement periods, pressures were measured simultaneously at the loudspeaker and tracheal ends of the wave tube with miniature differential pressure transducers (Honeywell Differential Pressure Sensor model 24PCEFA6D; Honeywell, Charlotte, NC, USA). Zrs was calculated as the load impedance of the wave tube [41]. At least four technically acceptable measurements were made at each stage of the protocol. The mechanical properties of the respiratory system were characterized by fitting a well-validated model [42] to the averaged Zrs spectra. The model comprised frequency-independent airway resistance (Raw) and airway inertance in series with a viscoelastic constant-phase tissue unit [42], and incorporated tissue damping (G) and elastance (H). Tissue histeresivity (η), which characterizes the coupling between the dissipative and elastic forces within the respiratory tissues was calculated as G/H [43].

### Measurement of intrapulmonary shunt fraction and oxygenation

For the blood gas analyses, 0.1-ml samples of arterial and central venous blood were collected simultaneously. The capillary (CcO_2_), arterial (CaO_2_), and venous (CvO_2_) oxygen contents were evaluated from the blood gases and used to calculate the intrapulmonary shunt fraction (Qs/Qt) by applying the modified Berggren equation [44]:

$$ \frac{Q_s}{Q_t}=\frac{CcO_2-{CaO}_2}{CcO_2-{CvO}_2} $$

To characterize the oxygenation efficiency of the lungs PaO_2_/FiO_2_ was calculated from the arterial blood gas assessments.

### Lung tissue histology

After completing the experimental protocol, a midline thoracotomy was performed and the lungs were fixed by introducing 4% formaldehyde via the tracheal cannula with a hydrostatic pressure of 20 cmH_2_O. The heart-lung blocks were then removed in one piece from the thoracic cavity. To visualize the collagen in the lung, tissue samples were fixed in 4% buffered formalin, embedded in paraffin, and 5-μm tissue sections were stained with Picro Sirius Red staining (Sigma-Aldrich). These were scanned with a Zeiss Mirax MIDI slide scanner at a magnification of × 20. At least three representative 0.15-mm^2^ sized rectangular regions of interest that contained alveoli without bronchi or large vessels were analyzed in each section. The collagen was segmented and quantified in the lung tissue sections by using the Trainable Weka Segmentation plugin in Fiji software [45]. The histological analyses were made by one person who was blinded for the group allocation.

### Experimental protocol

The experimental protocol is summarized in Fig. 1b. Following a 12-week housing period after the induction of DM (at the age of 20 weeks), each rat was anesthetized and the trachea was secured. The FRC was measured, followed by the surgical insertion of the arterial and venous catheters. Animals were initially ventilated with a PEEP of 3 cmH_2_O. A hyperinflation maneuver was then performed to standardize the volume history. After 3 min, arterial and venous blood gas samples were taken simultaneously and the first set of Zrs data was collected. Blood gas analyses and Zrs recordings were made at PEEP levels of 0 and 6 cmH_2_O, in random order. A period of 3 min was allowed for the animal after each PEEP step to maintain a steady state condition during the data collection. After completing the study on characterizing the PEEP-dependence, the PEEP was fixed at 3 cmH_2_O and a set of Zrs data was collected to establish the baseline for the bronchoprovocation tests with doubling doses (2, 4, 8, 16, and 32 μg/kg/min) of i.v. methacholine (MCh). At each MCh dose, steady-state bronchoconstriction was established (defined as < 5% difference in Raw between consecutive measurements) and forced oscillation Zrs data were measured. After completing the measurement protocol, each animal was euthanized by an overdose of pentobarbital and the lungs were removed for histological analysis.

### Statistical analyses

Data are expressed as mean ± standard deviation (SD) for normally distributed variables, and median with interquartile range (1st - 3rd quartile) otherwise. The Kolmogorov–Smirnov test was used to test data distributions for normality; where necessary, a logarithmic transformation was performed to normalize the data. Two-way repeated-measures analysis of variance (ANOVA) with Holm–Sidak post hoc analyses was used to assess the effects of the PEEP and group allocation on the respiratory mechanical and oxygenation parameters. Between group differences in airway responsiveness to MCh were evaluated with further two-way repeated-measures ANOVA with Holm–Sidak post hoc analyses. The MCh doses that elicited a 50% increase in Raw relative to baseline (PD_50_) were determined by fitting linear models to the individual dose–response curves. One-way ANOVA with Dunn’s post hoc analyses was used to evaluate the differences between groups in body weights, blood glucose levels, specific airway and respiratory tissue parameters, PD_50_, and the results of the histological analyzes. Correlation between the collagen expression in the lung tissue and viscoelastic properties of the respiratory system was determined by the Pearson correlation analysis. A sample size estimation for the repeated-measures ANOVA for the variable Raw, with a power of 0.8 and alpha of 0.05, indicated that at least 10 animals were required in each group to detect a statistically significant difference [46]. The statistical tests were performed with a significance level of *p* < 0.05.

## Results

Body weights, serum glucose levels, and the initial lung function parameters are summarized in Table 1. At the end of the 12-week treatment period, the mean body weight of the rats in the DM1 group was significantly lower than those in the DM2 and control groups. Significant elevations in blood glucose level were observed in the rats of the DM1 and DM2 groups compared to the control animals. The FRC measurements showed a significant reduction in static lung volume in the DM1 group rats; however, when the FRC values were normalized to body weight, they were significantly higher for the DM1 group rats than for those in the other two groups. Specific airway and respiratory tissue parameters were significantly higher in the DM1 and DM2 groups than in the control group.

**Table 1:** Main outcome parameters

	Group C (***n*** = 14)	Group DM1 (***n*** = 13)	Group DM2 (***n*** = 14)	p
**Body weight (g)**	563 ± 49	348 ± 91^*^	529 ± 76^#^	< 0.001
**Blood glucose (mmol/l)**	5.8 (5.6–6.1)	29.9^*^ (25.8–35.0)	14.75^*^ (7.0–27.3)	< 0.001
**FRC (ml)**	4.3 ± 0.5	3.6 ± 0.6^*^	4.4 ± 0.8^#^	0.003
**FRC**_**N**_**(ml/kg)**	7.5 (6.8–8.7)	10.2^*^ (8.6–1.3)	8.1^#^ (6.7–9.3)	0.003
**SRaw (cmH**_**2**_**O.s)**	0.23 (0.19–0.24)	0.27^*^ (0.24–0.32)	0.27^*^ (0.23–0.34)	0.004
**SG (cmH**_**2**_**O)**	3.1 (2.7–3–3)	3.4^*^ (3.2–4.0)	3.6^*^ (3.0–4.4)	0.023
**SH (cmH**_**2**_**O)**	12.0 ± 2.7	17.3 ± 4.9^*^	17.2 ± 5.8^*^	0.007

Main outcome parameters in the control rats (Group C) and in model of type 1 (Group DM1) and type 2 (Group DM2) diabetes mellitus presented as mean ± SD (Body weight, FRC and SH as normally distributed variables) and interquartile range (Q1 - Q3; Blood glucose, FRC_N_, SRaw, SG). FRC: functional residual capacity, FRC_N_: functional residual capacity normalized to body weight (FRC/BW), SRaw: specific airway resistance (Raw × FRC), SG: specific respiratory tissue damping (G × FRC), SH: specific airway resistance (H × FRC); all obtained without applying positive end-expiratory pressure. p: results of the one-way ANOVA tests and ANOVA on ranks. *: *p* < 0.05 vs. Group C, #: *p* < 0.05 between Groups DM2 and DM1 by using Dunn’s post-hoc analyses

**Fig. 2:**
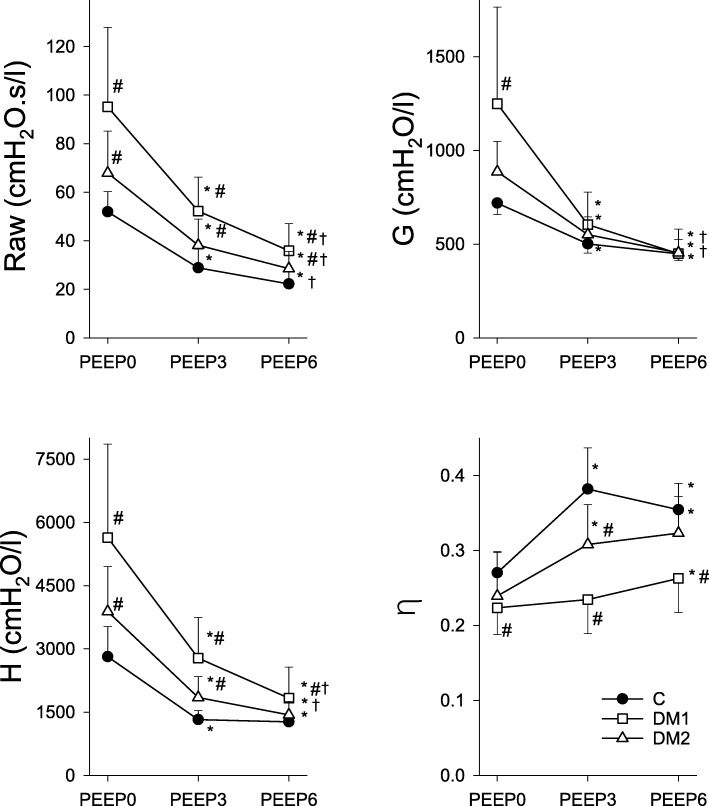
Airway and viscoelastic respiratory tissue mechanical parameters. Measurements obtained at positive end-expiratory pressures of 0, 3, and 6 cmH_2_O (PEEP0, PEEP3, and PEEP6, respectively) in control rats (closed circles, C, *n* = 14) and in rats in the type 1 diabetes (DM1, open squares, *n* = 13) and type 2 diabetes (DM2, open triangles, *n* = 14) groups. The symbols and error bars represent the mean and SD values, respectively. * *p* < 0.05 vs. PEEP0 within a group; † *p* < 0.05 vs. PEEP3; # *p* < 0.05 vs. C within a PEEP. Abbreviations: Raw, airway resistance; G, tissue damping (resistance); H, tissue elastance; η, tissue histeresivity (= G/H)

Figure 2 shows how the airway and viscoelastic parameters of the respiratory tissues varied with the PEEP for the three groups. Raw was significantly greater in both groups of diabetic animals than in the control group at all PEEP levels (*p* < 0.001). Two-way ANOVAs showed significant interactions between group and PEEP level for the parameters G (*p* < 0.001) and H (*p* < 0.001), indicating that the treatment affected these parameters’ dependence on PEEP. The dissipative properties of the respiratory tissues reflected by G were significantly compromised in the DM1 group when PEEP = 0 cmH_2_O. The respiratory tissue stiffness was elevated in both the DM1 (*p* < 0.001) and the DM2 (*p* = 0.039) groups at all PEEP levels, with more pronounced differences at low lung volumes. The dissociated PEEP dependences of G and H resulted in changes in η that varied between the groups, with significant decreases observed in the DM1 group for all three levels of PEEP (*p* < 0.001) and in the DM2 group when the PEEP was 3 or 6 cmH_2_O (*p* = 0.014) compared to the control group.

**Fig. 3:**
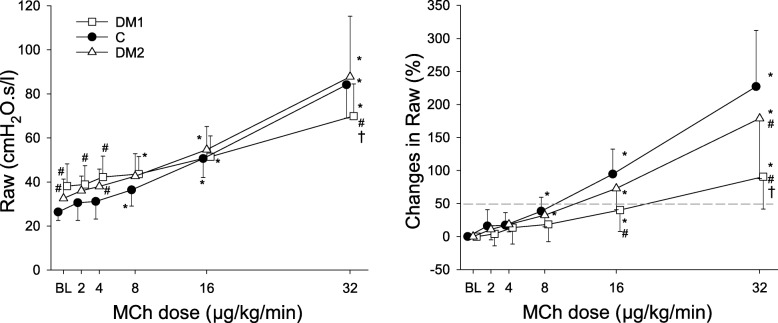
Airway resistance (Raw, left) and its changes relative to the baseline (BL) values (right) during the methacholine provocation tests in control rats (closed circles, C, *n* = 14), in rats in the type 1 diabetes (DM1, open squares, *n* = 13) and type 2 diabetes (DM2, open triangles, *n* = 14) groups. The symbols and error bars represent the mean and SD values, respectively. * *p* < 0.05 vs. BL within a group; # *p* < 0.05 vs. C within a condition; † *p* < 0.05 vs. DM2 within a condition

Airway resistance (left) and its changes relative to the baseline (right) during the MCh provocation tests are shown in Fig. 3. Significant elevations in the basal Raw values were observed in the DM1 (*p *< 0.001) and DM2 (*p* < 0.05) groups. These differences remained at the lower doses of MCh, whereas the absolute value of Raw became significantly lower in the DM1 rats at the highest dose of MCh (*p* < 0.05). Due to the significantly higher baseline Raw values in the diabetic rats, the differences in the dose-response curves to MCh between the protocol groups is more obvious when the responses are expressed as relative changes to baseline (right). In the control and DM2 groups, elevations in Raw were statistically significant from the 8 μg/kg/min MCh dose (*p* < 0.05), whereas in the DM1 group this increase was only observed after 16 μg/kg/min (*p* < 0.05). At the highest MCh dose, the MCh-induced relative increases in Raw were significantly lower in both DM1 and DM2 groups compared to control (*p* = 0.001). The characteristic shifts in the dose–response curves showed that the PD_50_ for MCh was significantly higher for the DM1 group (25.3 ± 20 μg/kg/min) than for the control group (8.9 ± 3.3 μg/kg/min, *p* = 0.001) and the DM2 group (12.7 ± 7.4 μg/kg/min, *p* = 0.026).

**Fig. 4:**
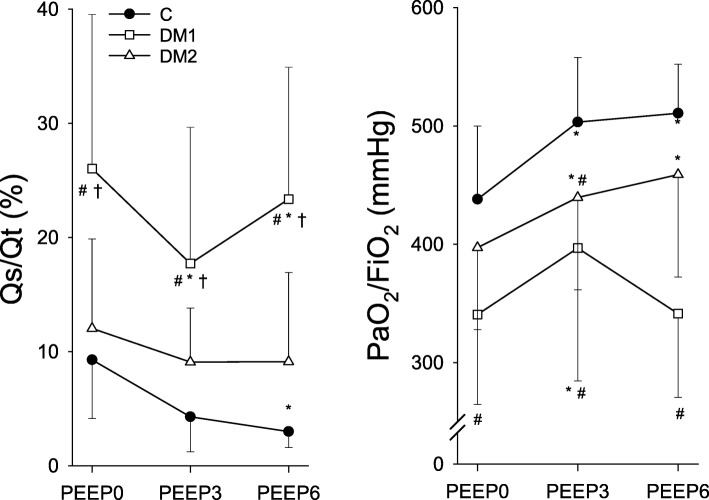
The intrapulmonary shunt fraction (Qs/Qt) and (PaO_2_/FiO_2_). Measurements obtained at positive end-expiratory pressures of 0, 3, and 6 cmH_2_O (PEEP0, PEEP3, and PEEP6, respectively) in control rats (closed circles, C, *n* = 14) and in rats in the type 1 diabetes (DM1, open squares, *n* = 13) and type 2 diabetes (DM2, open triangles, *n* = 14) groups. The symbols and error bars represent the mean and SD values, respectively. * *p* < 0.05 vs. PEEP0 within a group; # *p* < 0.05 vs. C within a PEEP; † *p* < 0.05 vs. DM2 within a PEEP

The parameters reflecting the gas exchange properties of the lungs are summarized in Fig. 4. The rats with healthy lungs in the control group exhibited moderate values of intrapulmonary shunt (< 9.3%) and physiological PaO_2_/FiO_2_ (> 438 mmHg); these indices exhibited systematic improvements with increasing PEEP to 3 (*p* < 0.02) and 6 cmH_2_O (*p* < 0.05). In the DM2 group, there was a near-significant tendency for Qs/Qt to be reduced compared to the control group at a PEEP of 6 cmH_2_O (*p* = 0.065), whereas PaO_2_/FiO_2_ was reduced at a PEEP of 3 cmH_2_O (*p* = 0.05) and tended to diminish at a PEEP of 6 cmH_2_O (*p* = 0.069). In the DM1 group at all three PEEP levels, there were significant increases in the intrapulmonary shunt (*p* < 0.001) associated with markedly compromised PaO_2_/FiO_2_ (*p* < 0.001). In addition, changes in the PEEP dependence of the gas exchange parameters were observed in the DM1 group, with no monotonous improvements in Qs/Qt and PaO_2_/FiO_2_ with increasing PEEP.

**Fig. 5:**
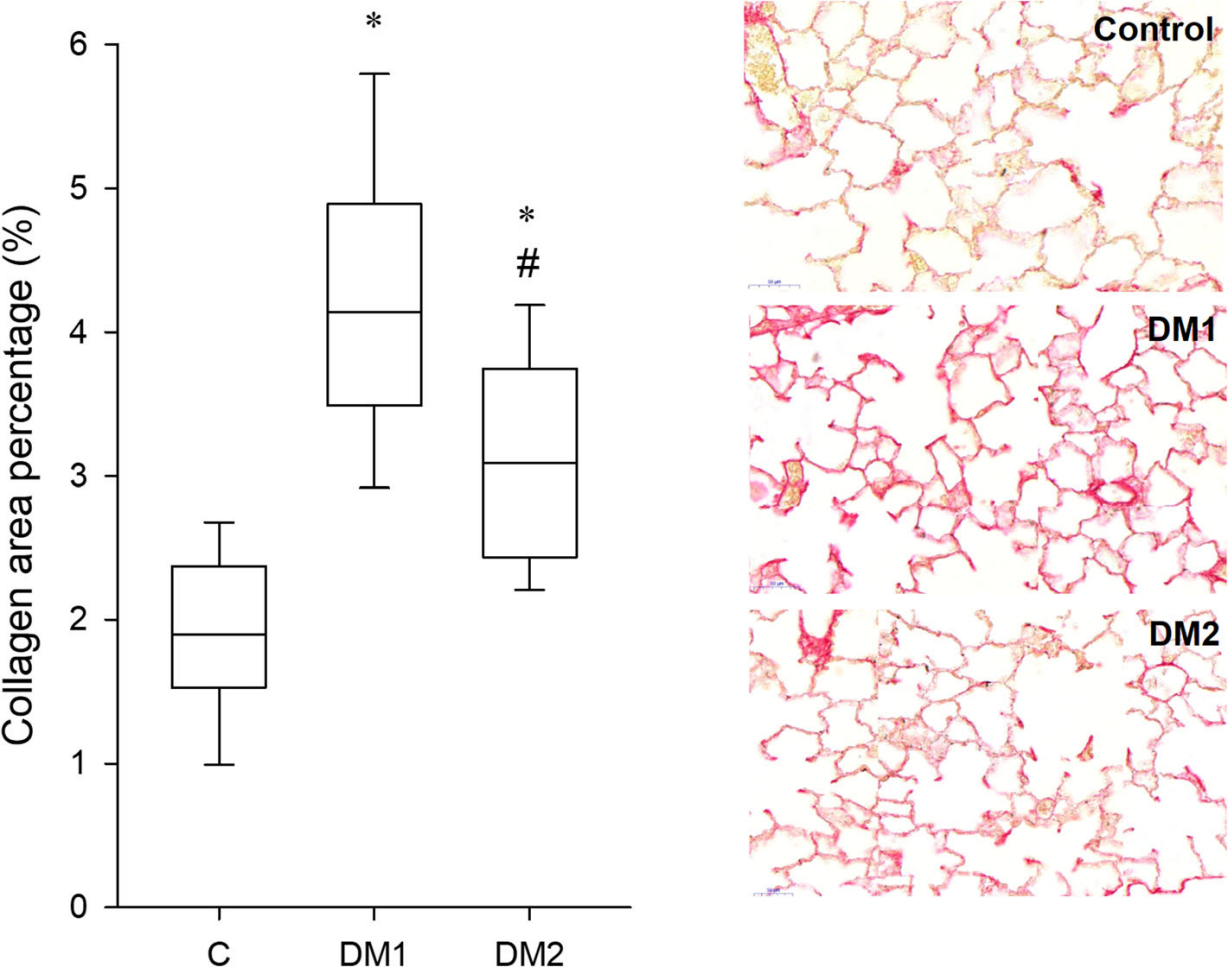
Collagen expression in the lung. Areas of collagen obtained from lung histology (left) and representative lung tissue sections in control rats (C, *n* = 14) and in rats in the type 1 diabetes (DM1, *n* = 13) and type 2 diabetes (DM2, *n* = 14) groups. * *p* < 0.05 vs. C; # *p* < 0.05 vs. DM1

Figure 5 shows the expression of collagen in the three groups. The percentage area of collagen was significantly higher in both DM groups than in the control group (*p* < 0.001). The collagen content of the lung parenchyma was greater in the DM1 group than in the DM2 group (*p* < 0.001).

**Fig. 6:**
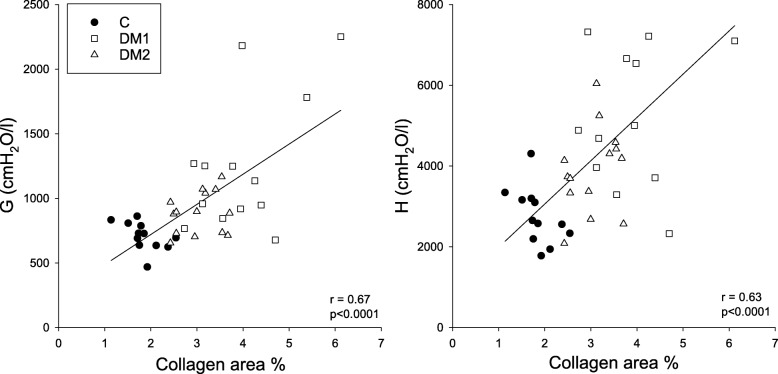
Correlation between the collagen and the viscoelastic parameters of the lung. Correlations between the percentage areas of collagen obtained by lung histology and forced oscillation mechanical parameters for tissue damping (G) and elastance (H) in control rats (C, *n* = 14) and in rats in the type 1 diabetes (DM1, *n* = 13) and type 2 diabetes (DM2, *n* = 14) groups. The solid line indicates the linear regression curve

Figure 6 shows the relationship between the percentage area of collagen obtained by lung histology and the viscoelastic dissipative and elastic mechanical parameters. There were significant correlations between the histology and both tissue mechanical indices, G and H, with correlation coefficients of 0.67 (*p* < 0.001) and 0.63 (*p* < 0.001), respectively.

## Discussion

The characterization of the pulmonary effects of DM in this study revealed detrimental changes in airway function, viscoelastic tissue mechanics, gas exchange, and collagen expression in the lung, with more severe manifestations in the rat model of type 1 DM than in that for type 2 DM. The increase in the basal airway tone with DM was associated with compromised dissipative and elastic tissue mechanics. The model of type 1 DM also showed diminished airway responsiveness to an exogenous cholinergic stimulus. These adverse mechanical and functional changes were accompanied by an increased intrapulmonary shunt and impaired PaO_2_/FiO_2_. Increasing the lung volume had a beneficial effect on the lung mechanics in both diabetic groups, whereas it had no benefit on gas exchange.

This study used well-established models to induce type 1 and 2 DM [34–39]. Type 1 DM was induced by a single high dose of STZ to cause the destruction of the pancreas, whereas type 2 DM was induced by a low dose of STZ to cause diffuse degeneration of the pancreatic cells, combined with a high-fat diet. As a result of these treatments, the blood glucose levels in both DM groups were markedly higher after 12 weeks than those of the control group. The body weight of the type 1 DM group rats was reduced, which can be explained by insulin deficiency [47, 48]. As with other chronic complications of DM, the pulmonary complications would be expected to manifest in the late stage of the condition [12]. For this reason, a 12-week period was allowed for the animals to develop lung dysfunction. This long period with markedly elevated serum glucose levels allowed the development of adverse pulmonary symptoms without a fatal outcome.

Forced oscillatory assessment of airway mechanics showed that DM caused a deterioration of basal Raw, with more severe changes occurring in the type 1 DM model rats (Fig. 2 PEEP0). As these changes were also apparent in the specific airway resistance values (Table 1) of both DM groups, the differences in lung size cannot solely be accounted for by the compromised airway mechanics. Instead, these pathological changes in the airways may be explained by the decreased vagal tone [49], excessive mucus production [50], low-grade chronic inflammation [51], activation of inflammatory pathways [52], or bronchial smooth muscle cell proliferation [5, 53]. The present study’s finding of increased airway tone in the DM rat models is in qualitative agreement with the results of previous studies that assessed airway resistance in DM [27, 54], providing supporting evidence for the potential development of chronic airway obstruction in patients with DM.

The effects of DM on the mechanical properties of the lung tissue has been studied in an ex-vivo experiment, and the results were limited to assess the elastic behavior of the lung parenchyma [55] However, dissipative properties of the lung tissue play an important role in determining the physiological lung mechanics and the changes of this component is characteristic in various lung diseases [56]. In the present study using an in-vivo setting, significant deteriorations were observed in the viscoelastic mechanical parameters of the respiratory tissues in both groups of diabetic rats (Fig. 2), and this adverse change affected both the dissipative and elastic properties. These differences compared to the control group remained after normalization of the values to the FRC (Table 1), indicating that intrinsic changes to the dissipative and elastic properties of the respiratory tissues can be anticipated. Collagen is the main determinant of overall lung tissue viscoelasticity [57, 58]. In the present study, the volume of collagen increased in the DM models, in agreement with results reported for patients with DM [59] (Fig. 5). There were significant correlations between the histological and mechanical findings (Fig. 6), which suggested that the overexpression of collagen may be a primary cause of the compromised tissue damping and elastance observed in DM. The underlying pathophysiological mechanisms responsible for this extracellular matrix remodeling may be related to the activation of pathological pathways that contribute to the formation of AGEs, which also stimulate the production of extracellular matrix components, including collagens, thereby affecting the interactions of the extracellular matrix [1].

Increasing numbers of patients with DM require surgical procedures that involve mechanical ventilation, which has increased the burden on healthcare providers [60, 61]. There is, therefore, a need for awareness that adequate mechanical ventilation should be provided for these patients. Open lung strategies require the application of an appropriate PEEP; however, the impact of different levels of PEEP on respiratory function in DM has not been clarified. Our findings demonstrated that the between-group differences in the airway resistance and in the tissue mechanical parameters representing viscoelastic dissipation and elastance disappeared at a moderately elevated PEEP (6 cmH_2_O; Fig. 2). The excessive PEEP dependence of the respiratory mechanical parameters was consistent with the diminished surfactant function reported previously in models of DM [21, 22]. It suggests that applying PEEP can have a beneficial effect on respiratory mechanics in this metabolic disease. The mechanical improvements were reflected in the decreased intrapulmonary shunt and increased PaO_2_/FiO_2_ in the DM rats when a PEEP of 3 cmH_2_O (Fig. 4) was applied. Nevertheless, substantial differences remained even with elevated PEEP in both groups of DM rats; this can be attributed to the persistent alveolar-capillary barrier damage observed in DM [26, 59, 62]. The type-II pneumocyte and surfactant layer damage [21, 22] along with the low-grade inflammation [26] observed in DM can contribute to the alveolo-capillary dysfunction. Noticeably, there was recurrent deterioration of PaO_2_/FiO_2_ and Qs/Qt in the type 1 DM rats at a PEEP of 6 cmH_2_O. This group would be expected to develop the well-established adverse pulmonary vascular consequences of hyperglycemia [63]. Accordingly, formation of AGEs as detailed above contributes to a proliferation of pulmonary endothelial and vascular smooth muscle cells and thicker basal lamina [64], which may have resulted in the intra-acinar and alveolar arterioles becoming prone to collapse when the PEEP exerted an external mechanical load on the capillary network.

An important physiological feature of airways is the adaptation of their caliber in response to exogenous stimuli. The results for the type 1 DM group demonstrated diminished airway responsiveness to MCh, which indicated that the adaptation ability of the bronchomotor tone had been severely compromised. Pathophysiological mechanisms that may have been involved in the decreased airway responsiveness include compromised vagal tone development due to autonomic diabetic neuropathy [49, 65], smooth muscle cell dysfunction [66], and/or epithelial damage [13]. Hyperinsulinemia, insulin resistance and hyperglycemia can lead to hyperproliferation and phenotype changing of the airway and bronchial smooth muscle cells and induce tracheal wall thickening [5, 53]. The smooth muscle cell damage might occur due to various factors, for instance disturbances in the nitric oxide synthesis [13], TGF-β and Rho-associated protein kinase pathway activation [16, 67, 68]. Nonetheless, conflicting results have been reported for the effects of DM on airway responsiveness. The findings of the present study are consistent with earlier findings of a reduction in the bronchial response to cholinergic stimuli in DM [13, 29, 69]. Previous reports of no change in airway responsiveness [28, 66, 70] or the development of airway hyperresponsiveness [16, 17, 71] may be explained by the insensitivity of the methods to assess airway function [66], the lack of airway innervation [16, 17, 70], or the involvement of confounding factors, such as, smoking, genetic differences and/or phenotype, and the duration of DM [28, 71]. Furthermore, in previous clinical studies, diabetes was often associated with comorbidities affecting the respiratory system, which may cause variation in the manifested pulmonary effects, and can contribute to the discordant results in the literature.

Several methodological aspects of the present study warrant consideration. As well-established models were adapted in the present study to produce the most important features of DM, including hyperglycemia and insulin deficiency, verification of the effectiveness of the STZ treatments was limited to evaluating serum glucose levels; a detailed characterization of the resulting metabolic profile would go beyond the focus of the present investigation. The models of type 1 and type 2 DM differed only in the dose of STZ and the diet regimen. The high dose of STZ administered to the rats in the type 1 DM group was likely to destroy most of the pancreatic β-cells, whereas less severe β-cell destruction in the type 2 DM group was complemented by a high-fat diet to induce a combination of impaired insulin production and peripheral insulin resistance [35, 48]. The difference in body weight between the groups suggested marked differences in their metabolic status; however, the respiratory consequences were analogous in the two DM groups, with symptoms differing only in severity. This study was designed to focus primarily on the changes in the mechanical properties of the respiratory system in diabetes. However, there are plethora of diagnostic tools to characterize pulmonary dysfunction both at on organ and at cellular levels. Analyses of bronchoalveolar lavage fluid to assess altered surfactant function, or quantitative collagen and smooth muscle cell assays by using immunohistochemistry methods would give further insights into the underlying mechanisms; these methods may be subjects of forthcoming investigations.

## Conclusion

In summary, distinct measurements of airway and respiratory tissue viscoelastic parameters in models of type 1 and type 2 DM showed evidence of detrimental changes in both compartments. The decline in airway function was reflected in elevated airway resistance and abnormal adaptation of the airways to exogenous constrictor stimuli. Lung tissue remodeling was manifested in compromised viscoelastic tissue mechanics, which affected dissipative and elastic properties, and the concurrent overexpression of collagen fibers in the extracellular matrix. These detrimental mechanical defects were overcome by applying PEEP, which demonstrated alveolar collapsibility in DM. However, even with the application of PEEP, gas exchange parameters remained compromised in the models of type 1 and type 2 DM, even deteriorated further in the type 1 DM model, indicating alveolo-capillary dysfunction. These findings may contribute to an improved understanding of the pulmonary consequences of DM and hold promise for the advancement of therapeutic interventions. PEEP titration during mechanical ventilation guided by respiratory mechanical parameters might be beneficial for this increasingly prevalent patient population.

## Data Availability

The datasets used and/or analysed during the current study are available from the corresponding author on reasonable request.

## Abbreviations

DM: Diabetes mellitus
ANOVA: Analysis of Variance
CcO_2_: Capillary oxygen content
CaO_2_: Arterial oxygen content
CvO_2_: Venous oxygen content
FiO_2_: Inhaled fraction of oxygen
FRC: Functional residual capacity
G: Lung tissue damping
H: Lung tissue elastance
MCh: Methacholine
PaO_2_: Arterial oxygen partial pressure
PaO_2_/FiO_2_: Arterial partial pressure to inspired oxygen fraction
PEEP: Positive end-expiratory pressure
Q1,Q3: First quartile and third quartile (respectively)
Qs/Qt: Intrapulmonary shunt fraction
Raw: Airway resistance
SD: Standard deviation
STZ: Streptozotocin
